# Assessing Knowledge of Pharmacokinetics in an Integrated Medical Curriculum

**DOI:** 10.1007/s40670-021-01442-4

**Published:** 2021-10-18

**Authors:** Rahul Pandit, Mirjam A. F. M. Gerrits, Eugène J. F. M. Custers

**Affiliations:** 1 Department of Translational Neuroscience, University Medical Center Utrecht (BCRM-UMCU), Utrecht University, Brain Center Rudolf Magnus Universiteitsweg 100, P.O. Box 85500, 3584 CG Utrecht, The Netherlands; 2 Center for Research and Development of Education, Utrecht University, University Medical Center, Utrecht, The Netherlands

**Keywords:** Pharmacokinetics, Medical education, Integrated medical curriculum, Teaching pharmacology

## Abstract

Pharmacokinetics is the branch of pharmacology that describes how the body processes drugs. As most physicians will prescribe drugs during their career, knowledge of pharmacokinetics is indispensable for medical students. Students, however, experience pharmacokinetics as difficult, probably due to its abstract and mathematical nature. In many medical curricula, pharmacokinetic topics are taught and examined as a part of integrated medical courses. As pharmacokinetics is a relatively small subject, unit examinations contain only few questions on the topic. The combination of a difficult subject and a few questions has raised concerns that students could perform poorly in pharmacokinetics and still pass the examinations and, hence, end up with insufficient knowledge of pharmacokinetics. In this study, we investigate this issue by contrasting students’ performance on pharmacokinetics questions with their performance on the rest of the examinations (all non-pharmacokinetics questions lumped together). The results expressed as pass-fail scores showed that students failed more often on the pharmacokinetics part of the test than on the other questions, in two consecutive academic years. Despite the suboptimal knowledge in pharmacokinetics, students can still acquire their bachelor’s degree. These results show that poor knowledge in pharmacokinetics could be a side effect of curricular integration. Attention should therefore be paid to provide insight into one’s own performance in individual disciplines. This would avoid knowledge deficiency and incompetence in the future.

## Introduction

Pharmacokinetics is the branch of pharmacology that studies the absorption, distribution, hepatic processing, and finally elimination of drugs; in short, how the body processes drugs. Each drug has a set pharmacokinetic profile in the body which ultimately determines the exposure of the body to the drug between administration and elimination. For example, age-related changes in the elderly or renal diseases can affect how drugs are processed in the body and would therefore influence the efficacy of pharmacotherapy. Such patients often require adjusted dose regimens or different drugs altogether [1, 2]. Since clinical guidelines cannot take into account all possible exceptions, the health sciences professional may have to adjust pharmacotherapy on a case-by-case basis. Without the proper knowledge of pharmacokinetics, prescribing errors might occur causing potential harm to the patient.

Despite its clinical relevance, many biomedical students experience pharmacokinetics as hard to grasp [3, 4]. Studies suggest that while studying pharmacology, biomedical students tend to focus more on the mechanism of action of drugs (pharmacodynamics), rather than pharmacokinetics [4, 5]. For example, in their study, Aronsson et al. [4] found that final-year medical and nursing students had difficulties understanding pharmacokinetic concepts and applying them in a clinical context. This could be due to the mathematical nature of the topic [3]. Pharmacokinetics requires reasoning and students have to rely on their theoretical knowledge to explain an observed event or case [6]. Although several studies mention pharmacokinetics as a potential stumbling block for biomedical students [3, 4, 6], it has not been systematically studied whether medical students indeed have poor knowledge of this topic. A lack of theoretical knowledge would make it difficult for students to solve problems that require reasoning in a clinical setting.

The integrated curriculum combines preclinical and clinical disciplines in thematic units to promote learning of preclinical disciplines in a clinical context, where application of this knowledge occurs [7]. In the integrated curriculum, student knowledge is usually tested as a part of a final unit test encompassing various disciplines [7]. This contrasts with the traditional discipline-based curriculum where preclinical disciplines such as anatomy, physiology, and pharmacology are taught as individual topics with independent final examinations. Although an integrated curriculum improves understanding of interrelationships between the individual disciplines [8], there is always the possibility that students do not master specific topics while they still pass the final unit test [9]. For example, there is evidence that students could deliberately study to achieve higher scores in other disciplines to compensate for lack of knowledge in clinical pharmacology [10]. This may hold *a forteriori* for pharmacokinetics, which is considered difficult by many students [4] and is usually only a small part of the unit test. In other words, medical students can pass examinations and acquire their bachelor’s diploma while ending up with insufficient knowledge of pharmacokinetics. The inherent difficult nature of the topic [4], combined with the fragmented method of teaching and modest number of hours dedicated to pharmacology topics in most medical curricula [11], makes this topic additionally vulnerable to be overlooked by students.

At the University Medical Center Utrecht (UMCU) Medical School, different pharmacokinetic topics are incorporated in three first-year courses (units) in the bachelor phase. Knowledge of pharmacokinetics is tested as part of a summative unit test at the completion of each unit. This implies pharmacokinetics is not taught as a single subject, but distributed over different units. The goal of the current study was to understand the impact of this fragmentation of pharmacokinetics teaching on overall student knowledge in this topic. To achieve this, we contrasted students’ scores in pharmacokinetics with scores in other disciplines pooled together.

Several medical curricula including ours require students to understand the mathematical relationships between pharmacokinetic parameters in order to calculate dose for drugs [12]. At the same time, students should be able to reason how pharmacokinetic parameters explain a certain phenomenon observed in a patient [6]. Based on these two sets of skills, pharmacokinetics can be divided into quantitative (requiring mathematical calculations) and qualitative parts (requiring reasoning or understanding of pharmacokinetic concepts). Therefore, as a secondary goal, we explored whether differences occur in student performance between these two categories of questions. This would provide further insight into the topics students find difficult in order to adapt teaching in the future.

## Method

### Study Population and Data Collection

Results from academic years 2016–2017 and 2017–2018 were retrospectively analyzed in the current study and students were taught the same curriculum and the same teacher conducted lectures and examinations. The first year of the medical curriculum at the UMC Utrecht comprises eight units of which three discuss various pharmacokinetic principles. A total of 14 h are dedicated to pharmacokinetics in the first year. Data of these three units were analyzed in the current study. Students participating in all three unit tests containing pharmacokinetics were included in the final analysis. Ethical approval for this study was obtained from the Dutch Association for Medical Education Ethical Review Board (NERB: 2017.7.2).

### Data Processing

Data from the digital examination platform (Testvision™) were extracted and analyzed as described below.

#### Virtual Examinations of Pharmacokinetics and Other Disciplines

Questions on pharmacokinetics across three units (A, B, and C) were pooled together (*pharmacokinetics*). These results were compared with the combined results of questions on other disciplines (*other disciplines*). Other disciplines included topics from anatomy, physiology, biochemistry, and histology. This is, in fact, a “virtual” examination which treats pharmacokinetics (in fact distributed over different examinations) *as if* it were a stand-alone examination. Furthermore, similar to other medical faculties [10], the UMCU Utrecht also uses an absolute cutoff score to distinguish between students who pass and students who fail on the examination. This cutoff score is set at 55% and takes the probability of guessing into account. The cutoff score therefore varies per examination and depends on the number of questions where guessing is a factor (i.e., on closed questions).

#### Question Characteristics

Questions were formulated by the lead lecturer of the various topics and were cross-checked for quality by at least one (senior) educator. Both closed-ended and open-ended questions were included in the analysis. The majority of closed-ended questions were assigned one point each, with a few exceptions where more points were assigned. Open-ended questions varied between one and ten points and usually consisted of a number of sub-parts. Partially correct answers for both open-ended and closed-ended received points accordingly.

#### Analysis of Exam Questions

Individual questions were further characterized by the difficulty (*p*-value) and discrimination (rir-value) index [13]. The program TestVision automatically calculates these values for each question and we used them in our study. The *p*-value for questions with one possible answer indicates the proportion of students who correctly answered the question. For questions with multiple possible answers or open-ended questions, the *p*-value indicates the proportion of the points obtained by students for a particular question [13]. In both cases, *p*-values range from 0 to 1 with easier items resulting in higher *p*-values. In order to understand whether the level of difficulty of these virtual examinations could explain the observed differences in student performance, the average *p*-values for the virtual examinations were compared. The second variable of interest, the rir-value, indicates the correlation between the performance of students in a question and the performance in the whole test excluding that question. These values vary between −1 and + 1. Positive rir values indicate that candidates scoring high in the test also perform well on that particular question [13]. Similarly, we calculated the average rir value for the virtual examinations to observe any differences in the discriminative index of the included questions. In contrast to open-ended questions where generally no guessing is possible as students have to formulate their own answer, closed-ended questions are associated with a certain degree of guessability. Students might simply choose an answer as multiple answers are provided. In order to take guessability into account, each test item is provided with a probability of guessing. Guessability is the number of points that a student can obtain by simply guessing the answer to a closed question, e.g., 0.25 points on an MCQ that contains four alternatives. To investigate whether the virtual examinations had differences in guessability, the average probability in guessing across two virtual examinations was calculated.

#### Determination of Student Performance in Pharmacokinetics vs Other Disciplines

Student performance was defined using two parameters: scores and a dichotomous pass-fail categorization. Firstly, for every student, we had two scores: the number of points she/he obtained on the pharmacokinetics questions of the three units and the corresponding score on the remaining questions. To make the scores comparable, they were expressed as percentages of the maximum number of points that could be obtained if a student answered all questions correctly. Secondly, to investigate the number of students passing the virtual examinations, the cutoff value was also applied to this score.

#### Distinction Between Qualitative and Quantitative Pharmacokinetics Questions

A further distinction was made between questions requiring reasoning of pharmacokinetic concepts (qualitative pharmacokinetics) vs. questions that required students’ performing mathematical calculations (quantitative pharmacokinetics). This is based on the two overall objectives of pharmacokinetics, namely understanding the interrelationship between the various pharmacokinetics parameters to apply them for solving a clinical problem and calculating drug doses. For the latter, during the unit tests, students are provided with a list of the necessary pharmacokinetic formulas and hence are not required to know these formulas by heart. Out of the 14 h dedicated to pharmacokinetics, approximately 5 h are dedicated to quantitative pharmacokinetics and the rest to qualitative pharmacokinetics. Examples of quantitative (1) and qualitative questions (2, 3) are provided in Box 1.

**Figure:**
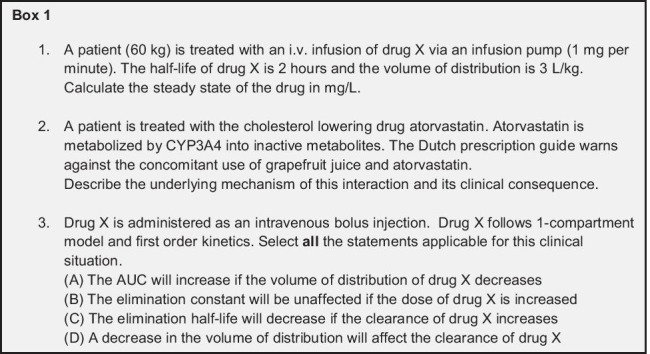

### Statistical Analyses

Average scores and standard deviations were calculated for examination parameters (*p*-value, rir-value, guessability) and student scores. Pass or fail decisions based on the test scores were considered as categorical variables and were represented as numbers of students (and percentages). Differences in scores between the two groups (pharmacokinetics vs other disciplines) were analyzed by Student’s *t*-test. Cohen’s *d* coefficient was used to determine effect size and was calculated online [14]. Differences in the number of students passing or failing corrected for guessing were analyzed by McNemar’s test. For all tests, a statistical *p*-value of 0.05 was considered significant.

## Results

### Characteristics of the Virtual Examinations Pharmacokinetics and Other Disciplines

Data were available for 290 (2016–2017) and 287 (2017–2018) students who took all three unit tests. Table 1 provides characteristics of the virtual examinations of pharmacokinetics and other disciplines. Unit A and B comprised of open- and closed-ended questions and unit C consisted solely of closed-ended questions. Pharmacokinetics was a small topic in the examinations and the total number of points dedicated to pharmacokinetics was lower than that of other disciplines. The average *p*-values for 2016–2017 were 0.76 ± 0.17 (pharmacokinetics) vs. 0.73 ± 0.21(other disciplines) and those of 2017–2018 were 0.77 ± 0.15 (pharmacokinetics) vs. 0.74 ± 0.17 (other disciplines). These values showed no significant difference (2016–2017: Student’s *t*-test, *p* = 0.439; 2017–2018: Student’s *t*-test, *p* = 0.439). The average rir-values for 2016–2017 were 0.21 ± 0.12 (pharmacokinetics) vs. 0.21 ± 0.12 (other disciplines). This was not statistically different (Student’s *t*-test, *p* = 0.867). The average rir-values of 2017–2018 were 0.26 ± 0.13 (pharmacokinetics) vs. 0.22 ± 0.12 (other disciplines) and were statistically not different (Student’s *t*-test, *p* = 0.302). Finally, the average guessability for the virtual examinations for 2016–2017 was 0.27 ± 0.10 (pharmacokinetics) vs. 0.25 ± 0.11(other disciplines), and those for 2017–2018 were 0.28 ± 0.15 (pharmacokinetics) vs. 0.27 ± 0.11 (other disciplines). The two virtual examinations did not differ in guessability for both years (2016–2017: Student’s *t*-test, *p* = 0.650; 2017–2018: Student’s *t*-test, *p* = 0.809).

**Table 1:** Characteristics of the virtual examinations for pharmacokinetics only or other disciplines. Rows A, B, and C indicate individual-unit tests where pharmacokinetics is taught. The number of points and number of questions (in brackets) are subdivided into open- and closed-ended categories. The row Totals indicates the total number of points and number of questions (in brackets) for the “virtual tests.” Cutoff score with corresponding percentages in brackets represents the minimum number of points needed to pass the virtual examination

	**Pharmacokinetics**	**Other disciplines**
**2016–2017**		
** A**	Closed-ended 4 (4) Open-ended 10 (1)	Closed-ended 44 (32) Open-ended 24 (5)
** B**	Closed-ended 2 (2)	Closed-ended 55 (55) Open-ended 3 (3)
** C**	Closed-ended 8 (8)	Closed-ended 42 (42)
** Totals**	Closed-ended 14 (14) Open-ended 10 (1) Combined 24 (15)	Closed-ended 141 (129) Open-ended 27 (8) Combined 168 (137)
** Cutoff score**	15 (62.5%)	107.9 (64.2%)
**2017–2018**		
** A**	Closed-ended 4 (4) Open-ended 11 (2)	Closed-ended 35 (31) Open-ended 30 (4)
** B**	Closed-ended 2 (2)	Closed-ended 54 (54) Open-ended 4 (4)
** C**	Closed-ended 8 (8)	Closed-ended 42 (42)
** Totals**	Closed-ended 14 (14) Open-ended 11 (2) Combined 25 (16)	Closed-ended 131(127) Open-ended 34 (8) Combined 165 (135)
** Cutoff score**	15.7 (62.8%)	107.1 (64.9%)

### Comparison of Student Performance in Pharmacokinetics vs Other Disciplines

For both academic years, a significantly higher number of students failed in pharmacokinetics compared to other disciplines (Table 2). Mean score for pharmacokinetics questions was significantly lower compared to other disciplines for the academic year 2016–2017. This tendency, however, was not found for the academic year 2017–2018 (Table 2).

**Table 2:** Comparison between student performance in pharmacokinetics only vs other disciplines combined expressed as pass-fail or obtained scores. Pass-fail data are expressed as number (%) and scores are expressed as mean ± SD

	**Pharmacokinetics**	**Other disciplines**	**Statistical test**
**2016–2017 (*N* = 290)**			
** Failed**	69 (23.8%)	31 (10.7%)	McNemar’s test, *p* < 0.001
** Score**	70.9 ± 14.2	74.1 ± 7.7	Student’s *t*-test, *p* < 0.001 Cohen’s *d* −0.6577
**2017–2018 (*N* = 287)**			
** Failed**	60 (21%)	40 (13.9%)	McNemar’s test, *p* = 0.007
** Score**	76.3 ± 15.2	73.3 ± 8.0	Student’s *t*-test, *p* < 0.001 Cohen’s *d* 0.5285

### Qualitative vs Quantitative Pharmacokinetics

Finally, we divided all questions on pharmacokinetics into two sub-categories qualitative and quantitative pharmacokinetics as shown in Table 3. Students on the average performed better on quantitative than on qualitative pharmacokinetics questions across both academic years (Table 3).

**Table 3:** Comparison between qualitative and quantitative pharmacokinetics questions for three units combined. For each year, the maximum number of points dedicated to the two sub-categories and the corresponding number of questions (in brackets) are provided. The row score (mean ± SD) indicates student performance in the two types of pharmacokinetics questions

	**Qualitative pharmacokinetics**	**Quantitative pharmacokinetics**	**Statistical test**
**2016–2017 (*N* = 290)**			
** Maximum number of points (questions)**	**18 (9)**	**6 (6)**	
** Score**	68.8 ± 16.3	77.0 ± 18.0	Student’s *t*-test, *p* < 0.001 Cohen’s *d* −0.7857
**2017–2018 (*N* = 287)**			
** Maximum number of points (questions)**	**21 (12)**	**4 (4)**	
** Score**	74.5 ± 16.2	85.8 ± 21.1	Student’s *t*-test, *p* < 0.001 Cohen’s *d* −1.0816

## Discussion

The primary aim of this study was to investigate whether students performed poorer on pharmacokinetics questions than on the remaining questions of the unit examinations. The current study is based on a large sample size (*n* = 287–290) and shows that a wide variation exists in students’ knowledge concerning pharmacokinetics. If our regular pass-fail standards were applied, approximately one in four or five students would fail in pharmacokinetics if this topic was examined as a stand-alone test . These figures are almost twice as high when compared to other disciplines pooled together and are consistent across two academic years. Furthermore, when looking at the scores, students obtained lower scores in pharmacokinetics compared to other disciplines in 2016–2017. Although this phenomenon was reversed in the academic year 2017–2018, still a higher failure rate in pharmacokinetics was observed for this academic year. A plausible explanation for this phenomenon could be a wider variation in student knowledge of pharmacokinetics in the academic year 2017–2018 as reflected in the wider standard deviation of student scores. One could conclude that students competent in pharmacokinetics obtained a higher score thereby increasing the average score of the group. However, when a pass-fail cutoff score was applied, a substantial number of the students still failed the virtual pharmacokinetics examination. As two variables (student scores and pass-fail rates) were used as outcomes, it is important to note that the average scores are not corrected for guessing. Since closed-ended questions are associated with a certain degree of guessability, it is important to include a correction factor. The cutoff score on the other hand corrects for guessing and might give a better indicator of the student performance.

The results of our study are similar to the findings of Wallerstedt et al. [10]. In this paper, the authors studied student performance in clinical pharmacology, another topic within pharmacology. The authors report low scores in clinical pharmacology, as compared to other disciplines when this topic was taught in an integrated curriculum. Our study reinforces the results obtained by Wallerstedt et al. [10] by suggesting that the insufficient knowledge in certain disciplines could be a wider phenomenon within the integrated curriculum. Furthermore, as our study indicates, this deficit in knowledge could start as early as the first year of medical school.

Could students leave aside pharmacokinetics while preparing for examinations? Given approximately 20% of total points in units A and C (Table 1) was dedicated to pharmacokinetics, it is highly unlikely that students would entirely skip pharmacokinetics while preparing for the individual-unit examinations. Therefore, the higher failure rates in the virtual pharmacokinetics examination reveal a side effect of curriculum integration, where the performance of individual subjects is not monitored. Furthermore, due to the fragmentation of pharmacokinetics teaching over several units, students could fail to notice their deficiency in this topic and continue their medical curriculum. This is a reason for concern and can be seen as a limitation of the integrated curriculum. Interpreting these results from the perspective of Miller’s Pyramid [15], our results show that one-fifth of medical students do not possess the required knowledge (*knows, knows how*) in pharmacokinetics, which would make application of this knowledge during clerkships (*shows*) or beyond (*shows how*) challenging. This trend in fact has been previously reported where final-semester health care students performed poorly when pharmacokinetics knowledge had to be applied in fictive clinical situations [4].

The higher failure rates in pharmacokinetics could depend on several factors such as the mathematical nature of pharmacokinetics [4] or the limited hours devoted to pharmacology as a subject [11]; but the lack of experience with prescribing might also play a role. Even though the virtual examinations in both cohorts had comparable indexes of difficulty, discrimination, and guessability, students still performed poorly in pharmacokinetics. This could imply that students find the topic pharmacokinetics intrinsically difficult, a finding also reported by others [5]. Supporting this theory, we find significantly lower scores on qualitative pharmacokinetics (requiring reasoning or understanding of pharmacokinetic concepts) questions than on quantitative pharmacokinetics questions (requiring mathematical calculations). These results are consistent over both academic years. The subject pharmacokinetics demands an understanding of abstract concepts in order to apply this knowledge in clinical settings [16]. Lower scores on qualitative questions that require reasoning suggest that students are unable to solve problems at a somewhat higher level of complexity, i.e., problems requiring analysis and application. However, they perform relatively well when they have to apply formulas to quantitative data. Although mathematical calculations are often seen as a stumbling block for students [3, 17], we did not observe this in our cohort.

The problems of fragmentation of pharmacology topics in an integrated medical curriculum have also been raised by other authors (Engels, 25). What are the implications of these findings for pharmacokinetics teaching in an integrated medical curriculum? Would our results support discipline-based teaching of pharmacokinetics in the medical curriculum? Based on our results, we however do not recommend separate examination of pharmacokinetics in the medical curriculum. Instead, we call for adapting the applied andragogic methods for teaching and examination. Like other universities [18], pharmacokinetics offered at UMCU uses a lecture and problem-based curriculum. Although these teaching methodologies are widely popular, individual accountability still remains a problem [19]. An alternative teaching method would be switching to team-based learning to encourage deeper understanding and application of concepts through peer engagement and by involving elements of immediate feedback [19, 20]. Team-based learning has been applied to teach pharmacology topics including pharmacokinetics and has been shown to have a positive effect on student knowledge [20, 21]. Moreover, switching to team-based learning does not necessarily need to occur at a curriculum level. The fact that individual discipline areas could initiate this switch [21], makes it a relatively accessible approach. Next to modifying methods of teaching, attention needs to be devoted to the method of testing. For example, reporting scores in individual disciplines could be a way to increase student insight into their own performance and competence [22]. Digital tools could help in segregating results of unit examinations into separate disciplines to provide a live summary of their performance in these disciplines [22]. This will additionally help instructors to adjust their teaching depending on topics students find difficult. Finally, another way to improve student knowledge in pharmacokinetics could be repetition. Repetition of pharmacokinetics knowledge in the clinical setting should be obligatory where the basic concepts of pharmacokinetics are discussed in a clinical context (clinical pharmacokinetics). Frequent repetition, distributed over time, especially in a simulated environment such as student-run clinics can stimulate student understanding especially when combined with immediate feedback [23, 24].

## Limitations of the Study

Although the current study sheds light on pharmacokinetics teaching within the UMCU and has helped us reflect and suggest teaching reforms, limitations of the study need to be mentioned. For example, the ideal way to investigate the effect of fragmentation of pharmacokinetics over several units would be to compare student performance in the integrated curriculum and a non-fragmented traditional discipline-based curriculum. Since all Dutch universities including ours have shifted to an integrated curriculum in the past decade, making such a comparison was impossible. Furthermore, we did not investigate individual preparation of students for the unit tests, and, hence, cannot be certain that students indeed put more effort into preparing for the non-pharmacokinetic parts of the examination in order to compensate for their deficient pharmacokinetic knowledge. Nevertheless, we have tried to address these issues by analyzing data across two academic years where students were taught the same curriculum by the same teacher conducting lectures and examinations. Another limitation is the difference in the total number of points in both groups for pharmacokinetics vs. other disciplines. This difference could underlie the greater spread in the pharmacokinetics group.

## Conclusion

A number of strategies such as clinical guidelines or electronic prescribing systems can be employed to minimize prescription errors. The latter can alert the prescriber of possible drug interactions or effects of diseased states on the pharmacokinetics. However, the implementation of these tools is not a replacement for the knowledge and reasoning the profession demands. Even experienced physicians who often use “pattern recognition” to solve clinical cases have to rely on their analytical skills when dealing with complex cases [24]. In everyday practice, failing to adjust treatment depending on the individual characteristics of the patient can have grave consequences for the patient. Some of these prescription errors could be prevented by optimizing pharmacokinetic education in the medical curriculum.

## Data Availability

The datasets generated during and/or analyzed during the current study are not publicly available due to privacy reasons but could be available from the corresponding author on reasonable request, provided prior permission is granted by UMC Utrecht and the Dutch Association for Medical Education Ethical Review Board.
